# Reduced Let-7f in Bone Marrow-Derived Mesenchymal Stem Cells Triggers Treg/Th17 Imbalance in Patients With Systemic Lupus Erythematosus

**DOI:** 10.3389/fimmu.2020.00233

**Published:** 2020-02-18

**Authors:** Linyu Geng, Xiaojun Tang, Shiying Wang, Yue Sun, Dandan Wang, Betty P. Tsao, Xuebing Feng, Lingyun Sun

**Affiliations:** ^1^Department of Rheumatology and Immunology, The Affiliated Drum Tower Hospital of Nanjing University Medical School, Nanjing, China; ^2^Division of Rheumatology & Immunology, Medical University of South Carolina, Charleston, SC, United States

**Keywords:** let-7f miRNA, systemic lupus erythematosus, bone marrow derived mesenchymal stem cell, interlukin 6, regulatory T cell (Treg), T help cell 17 (Th17)

## Abstract

Systemic lupus erythematosus (SLE) patients exist an imbalance between regulatory T (Treg) and T helper 17 cells (Th17), which might be contributed by defective immune regulation of bone marrow derived mesenchymal stem cells (BM-MSCs) from SLE patients. Our microRNA array analysis showed markedly down-regulated expression levels of microRNA let-7f in BM-MSCs from SLE patients compared to those from normal controls (NOR). To explore the role of let-7f in the disease pathogenesis, we showed that expression levels of let-7f in SLE BM-MSCs were negatively associated with SLE disease activity, and the predicted let-7 family targeted gene expression of interlukin-6 (IL-6) was significantly higher in BM-MSCs from SLE patients compared to normal controls (NOR). Transient transfection of BM-MSCs with let-7f mimics or inhibitors showed reduced levels of let-7f impaired the proliferation rate of BM-MSCs, BM-MSC-mediated downregulation of Th17 cells and upregulation of Treg cells, increased the apoptosis rate of BM-MSCs through targeting IL-6 and activating signal transducers and activators of transcription-3 (STAT3) pathway, but had no significant effect on the differentiation of Th1 and Th2. Our findings showed a key role of let-7f in the imbalance of Treg/Th17 mediated by SLE BM-MSCs, suggesting the potential of manipulating let-7f expression in BM-MSCs for treating SLE patients.

## Introduction

Accumulating evidences suggest that the imbalance of T-helper-cell (Th) subsets (Th1/Th2/Th17) and regulatory T-cells (Treg) contribute to the pathogenesis of systemic lupus erythematosus (SLE) (1–3), a systemic autoimmune disease characterized by disturbed T cell homeostasis. Recently, this imbalance in T cell homeostasis is thought to be contributed by defective immune regulation of bone marrow derived mesenchymal stem cells (BM-MSCs) from SLE patients (4, 5), but the underlying mechanisms still largely unknown.

Mesenchymal stem cells (MSCs), originally isolated from the bone marrow stroma, are multipotent and possess strong immunomodulatory functions which could modulate various immune disorders (6). MSCs can robustly interact with multiple immune cells, including dendritic cells (DCs), neutrophils, natural killer cells, T cells and B cells, thus these cells have been demonstrated as promising therapeutic benefits in various immune disorders. Previously, we showed that BM-MSCs from SLE patients exhibited abnormalities in modulating immune cells (7), and allogenic MSCs transplantation up-regulated Treg cells and down-regulated Th17 cells through the regulation of transforming growth factor β1 (TGF-β1) and prostaglandin E2 (PGE2) in lupus patients (8). However, the mechanisms for immune dysregulation of SLE BM-MSCs on Treg/Th17 cells are incompletely understood.

MicroRNAs (miRNAs) are single-stranded small non-coding RNAs, with ~19–23 nucleotides in length. In most cases, miRNAs act as negative post-transcriptional modulators of genes in eukaryotic organisms (9). Accumulating evidence shows that miRNAs, including miR-155, miR-146a, and miR-126, are actively involved in SLE pathogenesis through affecting the functions of T and B cells (10, 11). Recently, the roles of miRNAs in stem cell biology have aroused increasing attention (12–14). Our previous study demonstrated that increased miR-663 in SLE BM-MSCs contributed to lupus development through inducing imbalance of T follicular helper and T regulatory cells by targeting TGF-β1 and knock down of miR-663 in BM-MSCs may improve the efficacy of MSC-based cytotherapy (15). Besides miR-663, microRNA let-7 family especially let-7f has also been shown to be abnormally expressed in SLE BM-MSCs (15). Let-7 family is actively involved in diverse biological processes, including development, carcinogenesis, and autoimmunity, but the precise role of these miRNAs in lupus pathogenesis remains unclear. Considering of the miRNA functional similarity by miRNA functional networks study (16, 17), and the important role of let-7 family in immune system (18), together with the possible effect of let-7f in regulating TGF-β1 receptor 1 (TGFBR1) suggested by bioinformatics analysis, it is very likely that let-7f could be functionally similar to miR-663 or clustered as a functional sets with other miRNAs such as miR-663, which might also be involved in lupus pathogenesis mediated by regulating T cells.

Our microRNA array analysis showed markedly down-regulated expression levels of microRNA let-7f in SLE BM-MSCs compared to BM-MSCs from normal controls (NOR). Here we demonstrate that the reduced expression level of let-7f in SLE BM-MSCs is negatively associated with SLE disease activity. *In vitro* experiments confirm that let-7f reduction not only increases the apoptosis rate of BM-MSCs, but also impairs their function to downregulate Th17 cells and upregulate Treg cells through targeting interlukin-6 (IL-6), an important pro-inflammatory cytokine secreted by BM-MSCs (19). Thus, let-7f is a key mediator in SLE BM-MSCs regulation of Treg/Th17 and may serve as a promising therapeutic target to improve MSC-based cytotherapy for the treatment of lupus.

## Results

### Let-7f Level Is Decreased in SLE BM-MSCs and Negatively Associated With Disease Activity and 24 h Urine Proteinuria

**Figure 1:**
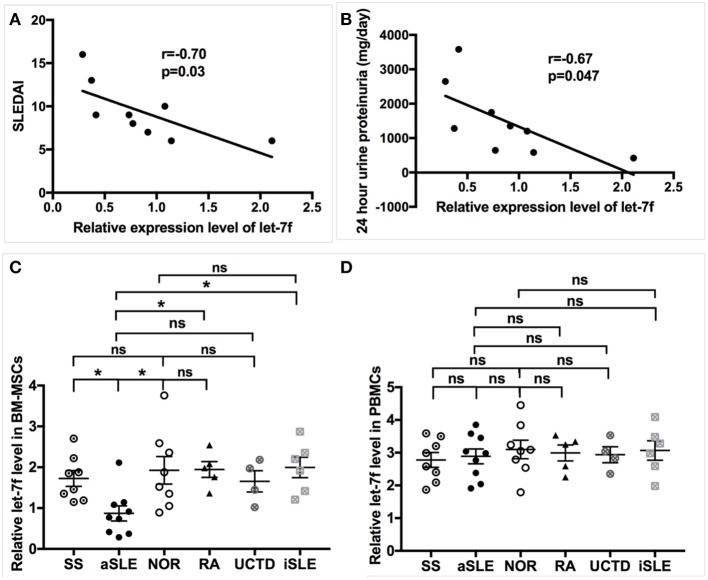
Association of let-7f in SLE BM-MSCs with clinical manifestiations of SLE patients. **(A,B)** Association of let-7f expression in BM-MSCs with SLE disease activity index (SLEDAI, **A**) score and 24 h urine proteinuria (mg/day, **B**). **(C)** Let-7f expression in BM-MSCs from patients with active SLE (aSLE, *n* = 9), inactive SLE (iSLE, *n* = 6), RA(*n* = 5), pSS(*n* = 8), UCTD (*n* = 4), and NOR (*n* = 10). **(D)** Let-7f expression in PBMCs from patients with aSLE (*n* = 9), iSLE(*n* = 6), RA(*n* = 5), pSS(*n* = 8), UCTD (*n* = 4), and NOR (*n* = 10). All data are mean ± SEM. *P < 0.05.

Our preliminary data have demonstrated several abnormally expressed miRNAs in BM-MSCs from SLE patients compared to those from normal controls (NOR) using human miRNAs arrays, among which the markedly reduced expression level of let-7f that was confirmed by qRT-PCR in BM-MSCs from SLE patients (15). Furthermore, levels of Let-7f expression negatively correlated with SLE disease activity index (SLEDAI, Figure 1A), 24 h urine proteinuria (Figure 1B) and erythrocyte sedimentation rate (ESR, Supplementary Table 4) significantly, supporting the potential involvement of let-7f in the pathogenesis of SLE, especially those with lupus nephritis. let-7f expression was only decreased in BM-MSCs from SLE patients with active disease, but not in BM-MSCs from inactive SLE patients and other connective tissue diseases (Figure 1C). In addition, no significant difference of let-7f expression was observed in peripheral blood mononuclear cells (PBMCs) from patients with SLE or other connective tissue diseases (Figure 1D), indicating an unique role of let-7f in SLE BM-MSCs.

### Let-7f Modulates IL-6 Expression in SLE BM-MSCs

**Figure 2:**
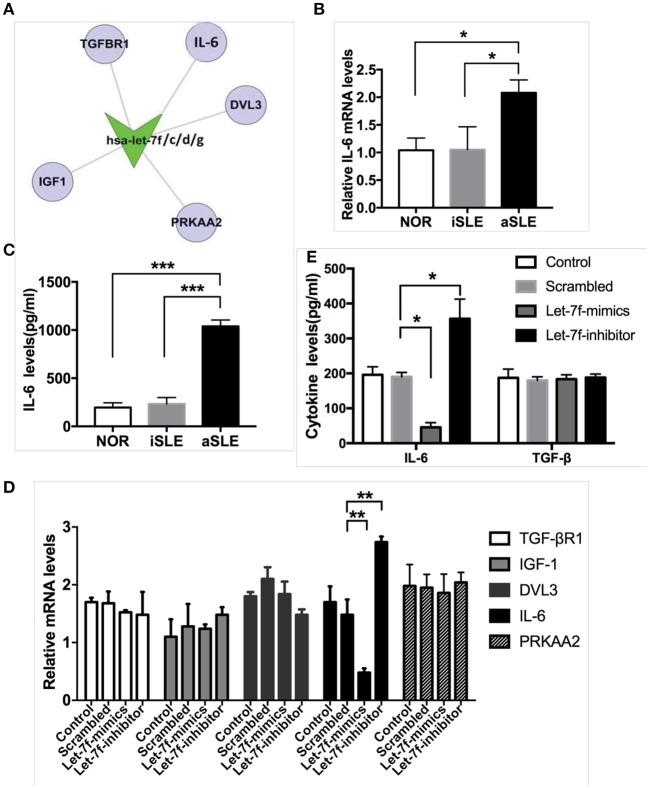
Let-7f could modulate IL-6 expression levels in BM-MSCs. **(A)** Predicted let-7 family targets by two computational methods, Targetscan and PITA. **(B)** IL-6 gene expressions in BM-MSCs from aSLE patients, iSLE patients and NOR. **(C)** aSLE BM-MSCs produce more IL-6 than that of BM-MSCs from NOR and iSLE patients after culturing for 24 h. **(D)** Gene expressions of predicted target of let-7 family in let-7f related oligonucleotide transfected BM-MSCs. **(E)** IL-6 and TGF-β protein levels in cultured supernatants of various let-7f related oligonucleotide transfected BM-MSCs. All data are mean ± SEM. For b-c, *n*(aSLE) = 9, *n*(iSLE) = 6, *n*(NOR) = 10; For d-e, *n* = 6. *P < 0.05, **P < 0.01, ***P < 0.001.

Next, we explored the underlying pathway regulated by let-7f. Using bioinformatics (Target Scan program and PITA), we identified IL-6 as predicted to be one of the putative targets of let-7f (Figure 2A). Previously, the NF-κB-Lin28-let-7-IL-6 positive feedback loop links inflammation to cancer and maintains cells at an epigenetic transformed state (19), suggesting that IL-6 is an important target gene of let-7f. IL-6 is also an important pro-inflammatory cytokine secreted by BM-MSCs. Our data showed that both mRNA (Figure 2B) and protein levels (Figure 2C) of IL-6 in SLE BM-MSCs were significantly elevated compared to those of healthy subjects, and IL-6 protein levels secreted by SLE BM-MSCs were negatively correlated with relative expression levels of let-7f in SLE BM-MSCs (*r* = −0.71, *p* = 0.03). To investigate whether let-7f could modulate the secretion of IL-6 by BM-MSCs, we overexpressed let-7f in BM-MSCs from healthy subjects by transfecting BM-MSCs with a synthetic let-7-mimics oligonucleotide (Let-7-mimic), or inhibited let-7f expression levels using anti-let-7f oligonucleotide (Let-7-inhibitor) complementary to mature let-7f sequences. Compared to non-transfected (Control) and scrambled control oligonucleotide transfected (Scrambled) BM-MSCs, Let-7-inhibitor transfection markedly increased IL-6 mRNA expression in BM-MSCs, while Let-7f-M led to an opposite effect, but had no effect on other putative target of let-7f including insulin-like growth factor 1 (IGF-1), protein kinase AMP-activated catalytic subunit alpha 2 (PRKAA2), disheveled segment polarity protein 3 (DVL3) and TGF-βR1 (Figure 2D). Similar to that of the mRNA level, with the downregulation of let-7f in BM-MSCs by Let-7f-inhibitor transfection, the protein levels of IL-6 protein levels in culture supernatant of BM-MSCs were increased (Figure 2E). Taken together, these data showed that let-7f could modulate IL-6 mRNA and protein levels in BM-MSCs from SLE patients.

### Let-7f Is Involved in BM-MSC Proliferation, Apoptosis, and Treg/Th17 Balance

**Figure 3:**
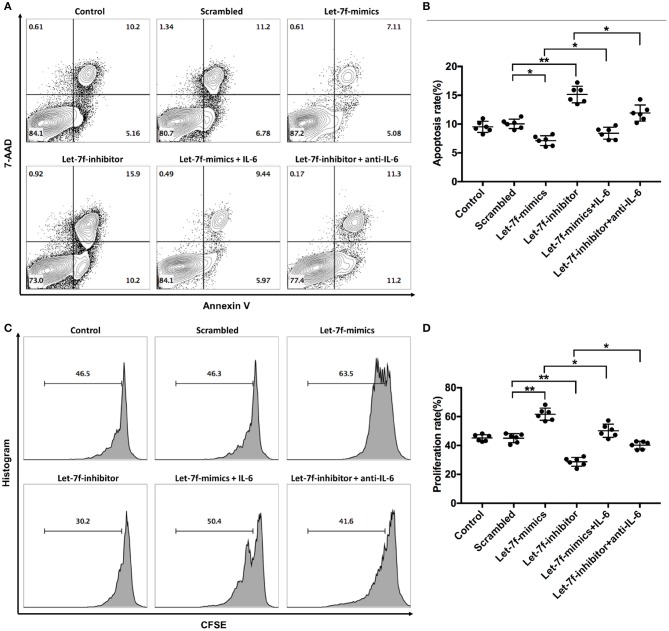
Let-7f affects BM-MSCs proliferation and apoptosis *in vitro*. **(A,B)** Apoptotic status of normal non-tranfected BM-MSCs (Control), normal BM-MSCs tranfected with let-7f scrambled oligonucleotides (Scrambled), Let-7-mimics, or Let-7-inhibitor with or without the presence of recombinant human IL-6 (50 ng/ml) or anti-human IL-6 antibody (30 μg/ml) by annexin V and 7-AAD staining assay. Dot plots showing the frequency of Annexin V^+^7-AAD^+^ late apoptotic cells of BM-MSCs among different groups. **(C,D)** Proliferation status of BM-MSCs among the six groups by CFSE assay. All data are mean ± SEM. *n* = 6, *P < 0.05, **P < 0.01.

**Figure 4:**
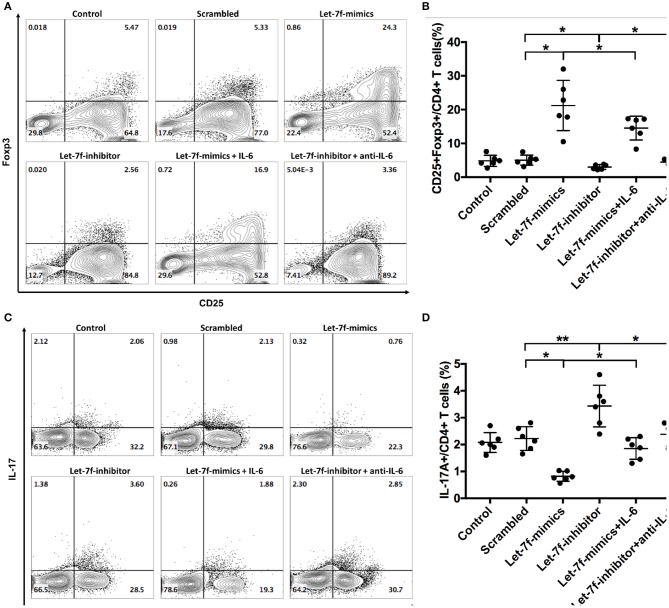
Let-7f upregulates immunoregulatory effects of BM-MSCs *in vitro*. **(A,B)** Percentages of T_reg_ cells (CD25^+^Foxp3^+^ /CD4^+^ T cells) after co-culturing of pre-stimulated naïve T cells with let7f-related oligonucleotide transfected BM-MSCs with or without the presence of recombinant human IL-6 (50 ng/ml) or anti-human IL-6 antibody (30 μg/ml). **(C,D)** Percentages of Th17 cells (IL-17A^+^/CD4^+^ T cells), after co-culturing of pre-stimulated naïve T cells with let7f-related oligonucleotide transfected BM-MSCs with, or without the presence of recombinant human IL-6 (50 ng/ml) or anti-human IL-6 antibody (30 μg/ml). All data are mean ± SEM. *n* = 6, *P < 0.05, **P < 0.01.

To investigated whether let-7f could modulate biological roles of BM-MSCs, cell functions including proliferation, apoptosis, and immune regulation were investigated after transfection of Let-7-mimics, Let-7-inhibitor, or Scrambled control oligonucleotide. Compared to scrambled control oligonucleotide transfected BM-MSCs (Scrambled), relative expression levels of let-7f were increased in Let-7f-mimics transfected BM-MSCs but decreased in Let-7f-inhibitor group 24, 48, and 72 h after let-7f related ectopic oligonucleotide transfection (Supplementary Figure 1A). Let-7f-mimics ectopic expression significantly promoted proliferation but suppressed apoptosis of BM-MSCs *in vitro*, while inhibition of let-7f by Let-7f-inhibitor transfection resulted in opposite effects (Figures 3A–D). To explore the effect of let-7f on immunoregulatory function of BM-MSCs, PBMCs from healthy controls were co-cultured with BM-MSCs transfected with let-7f related oligonucleotides for 3 days in the presence of anti-CD3/CD28 antibodies. Compared to scrambled oligonucleotide transfected BM-MSCs, Let-7f-mimics transfected BM-MSCs significantly increased the frequency of conventional Treg cells (CD25^+^Foxp3^+^ /CD4^+^ T cells, Figures 4A,B), but reduced the percentage of Th17 cells (IL17^+^/CD4^+^ T cells; Figures 4C,D), while let-7f-inhibitor transfected BM-MSCs downregulated the frequency of Treg cells but upregulated the frequency of Th17 cells (Figures 4A–D). Consequently, the ratio of Treg/Th17 was significantly upregulated in Let-7f-mimics transfected BM-MSCs while downregulated in Let-7f-inhibitor transfected BM-MSCs (Supplementary Figure 1D). However, no alteration of the Th1 and Th2 subsets was found after co-culturing with Let-7f-mimics or Let-7f-inhibitor transfected BM-MSCs (Supplementary Figures 1B,C). Thus, our data support that let-7f participate in BM-MSCs-mediated regulation of the Treg/Th17 cell ratio.

### Let-7f Affects BM-MSC Function via Inhibiting IL-6 Production

**Figure 5:**
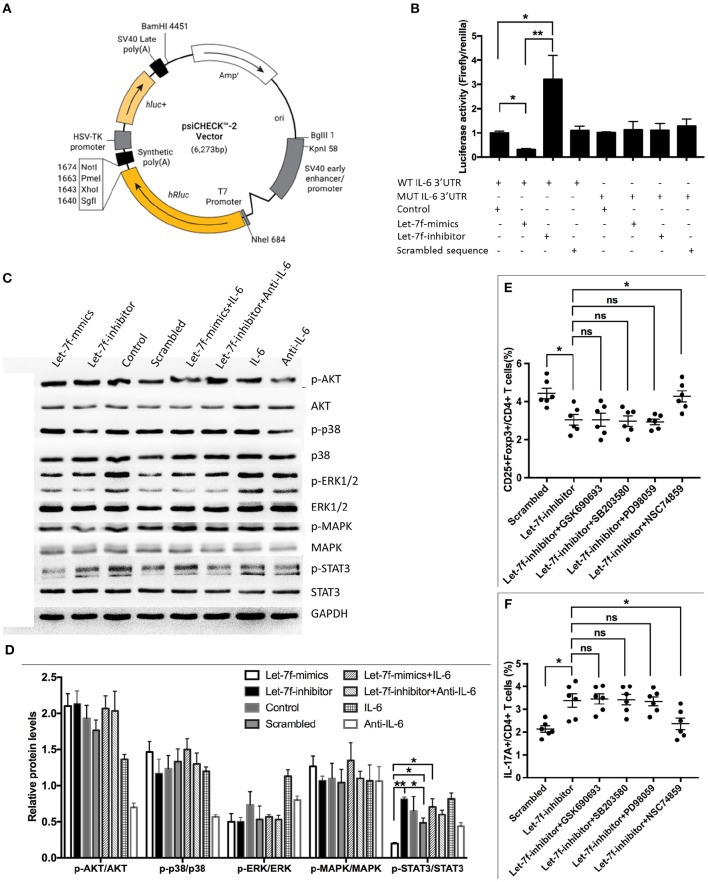
IL-6 is a direct target of let-7f. **(A)** Construction of TGF-β1'-UTR luciferase vector. **(B)** IL-6 response element reporter assay with let-7f precursor or let-7f inhibitors. IL-6 3′-UTR is involved in let-7f regulated gene expression. **(C,D)** The effect of let-7f and IL-6 on AKT, p38, ERK, MAPK, and STAT3 pathway in BM-MSCs. **(E,F)** The role of AKT inhibitor (GSK690693), p38/MAPK inhibitor (SB203580), and STAT3 inhi bitor (NSC74859) in immunoregulatory effect of let-7f related oligonucleotide transfected BM-MSCs on Treg and Th17 cells. All data are mean ± SEM. *n* = 6, *P < 0.05, **P < 0.01.

Considering that the abnormalities of let-7f suppressed BM-MSCs, including increased apoptosis, reduced proliferation and imbalance of Treg/Th17, were all reverted after suppression of IL-6 by anti-IL-6 antibody, while exogenous human recombinant IL-6 corrected Let-7f-mimics modified dysregulation of BM-MSCs (Figure 3), it is reasonable to consider that IL-6 plays an important role in let-7f regulation. To verify the direct binding of let-7f to IL-6 gene, we constructed the reporter plasmids containing either putative let-7f binding sites or mutated forms of the sites in 3′UTR region of IL-6 in BM-MSCs (Figure 5A). The luciferase activity of reporter plasmids containing a putative let-7f binding site of IL-6 3′UTR was downregulated, while the activity was not affected in the mutated form of IL-6 in Let-7f-transfected BM-MSCs (Figure 5B), confirming the specificity of the binding sequences. Taken together, our results demonstrated that IL-6 is a direct target of let-7f in BM-MSCs.

### STAT3 Signaling Pathway Is Involved in Impaired Let-7f Mediated Dysfunction of BM-MSCs

To gain further insight into the molecular mechanism, we investigated downstream cellular signals that could be initiated by IL-6, and participated in cellular events including protein kinase B (PKB, also known as AKT), signal transducers and activators of transcription-3 (STAT3) were investigated (20, 21). Interestingly, Let-7f-mimics transfected BM-MSCs significantly reduced phosphorylation of STAT3 in BM-MSCs but not AKT and p38/MAPK, while Let-7f-inhibitor led to an opposite effect. The effect of Let-7f-mimics could be abrogated by exogenous human recombinant IL-6 (Figures 5C,D), suggesting potential involvement of STAT3 in this process. Next, Let-7f-inhibitor transfected BM-MSCs were treated with AKT inhibitor (GSK690693), STAT3 inhibitor (NSC74859), and p38/MAPK inhibitor (SB203580). Consistently, compared to those without signaling pathway inhibitor, the addition of STAT3, but not of those of AKT or p38/MAPK, significantly upregulated the frequency of Treg cells and downregulated the frequency of Th17 cells in Let-7f-inhibitor BM-MSCs-PBMC co-cultures (Figures 5E,F). Thus, the STAT3 signaling pathway plays a critical role in impairing let-7f mediated dysfunction of BM-MSCs.

## Discussion

Many studies have shown the dysfunction of BM-MSCs in autoimmune diseases including lupus, which is critical for the acquisition and maintenance of immunological balance. Although genetic and/or epigenetic alterations in lupus pathogenesis are expected and widely characterized, little progress has been made in elucidating the molecular changes in the stroma of bone marrow and their potential functions. Recently, we identified a distinct miRNA signature in SLE BM-MSCs by miRNA array analysis (15). Here, we linked reduced expression levels of let-7f in SLE BM-MSCs to their impaired immunoregulatory functions, including the proliferation rate of BM-MSCs, BM-MSC-mediated downregulation of Th17 cells and upregulation of Treg cells as well as increases the apoptosis rate of BM-MSCs through targeting IL-6 and activating STAT3 pathway. These findings support a role of let-7f in lupus pathogenesis.

As the starting point of the RNA revolution, in the past decade, the discovery of let-7 miRNA, one of the first miRNAs discovered in the nematode, had aroused great interest for miRNA research work. Notably, the Lin28/let-7 regulatory network represents a critical signature in during pluripotency, reprogramming, and tumorigenicity (22, 23). Recently, let-7 has been demonstrated to be tightly associated with auto-immunity, specifically with toll-like receptors that mediate crucial roles in lupus development, which highlight its potential role as an efficient tool in lupus therapy. Chafin CB et al., have reported that increased expression levels of let-7a in the mesangial cells of pre-diseased and actively diseased NZB/W mice could increase the production of IL-6 *in vitro* (24). Liu et al., demonstrated that increased let-7a/e/i/g in kidney tissue samples of lupus nephritis (LN) patients could modulate the activation of NF-κB by targeting TNFAIP3, besides, lin28, a negative regulator of let-7 miRNAs, was found to be decreased in kidney samples from LN patients compared to the control samples, these results indicate a Lin28-Let-7-TNFAIP3 axis during the pathogenesis of LN (25), but none of these let-7 studies focusing on the bone marrow microenvironment in lupus pathogenesis, especially BM-MSCs. Here we demonstrated for the first time that lowering let-7f expression in SLE BM-MSCs might play a role in lupus pathogenesis through targeting IL-6. The reason for the opposite expression levels of let-7 in BM-MSCs from active SLE patients and kidney tissues from LN patients might be the followings: First, compared with peripheral blood and kidney tissue miRNA microarray analysis results (11), our previous data suggest that SLE patients may present a distinct BM-MSC miRNA signature compared with other tissues (15). In addition, the patients in the two groups have different inclusion criterial. The patients for microarray analysis in our groups were active lupus, while in their study, only patients with LN were included.

Among several let-7 family members, only let-7f was down-regulated in SLE BM-MSCs was identified by our micro-array assay and confirmed by RT-PCR assay. It is possible that other let-7 family subsets including let-7a, let-7b may reduce IL-6 secretion by BM-MSCs. However, ectopic expression of let-7a and let-7b had no effect on IL-6 mRNA levels (see Supplementary Figures 2A,B). To further validate whether the binding between let-7 subsets and IL-6 3′-UTR regulates the transcription of IL-6, luciferase reporters containing the wild type or mutated 3′-UTR of human IL-6 were co-transfected with let-7a or let-7b plasmids into HEK293T cell lines. Co-transfection of either let-7a-M/let-7a-I or let-7b-M/let-7b-I did not alter the luciferase reporter activity (see Supplementary Figures 2C,D), supporting that let-7a or let-7b could not regulate IL-6 expression through binding to the IL-6 3′-UTR.

Treg and Th17 cells, which share a common precursor cell (the naïve CD4 T cell), require a common TGF-β signal for initial differentiation (1, 26). However, terminally differentiated cells fulfill opposite functions: Th17 cells cause autoimmunity and inflammation, whereas Treg cells inhibit these phenomena and maintain immune homeostasis (1, 2). Emerging studies about patients and experimental models have determined the imbalance between Th17 and Treg cells as well as the related cytokines in the serum, including IL-6, IL-1b, and IL-23, and signal transducer, participated in the onset and progression of autoimmune diseases including SLE (3, 27), immune therapy could restore Treg/Th17 imbalance and thus ameliorate SLE and RA (4, 8, 28–30). Considering of the important immune regulatory effect of stem cells on Treg/Th17 (8, 15, 31), the imbalance between Treg and Th17 cells might be associated with SLE BM-MSC dysfunction. In the present study, overexpression of let-7f in BM-MSCs led to increased Treg and decreased Th17 cells, supporting that targeting let-7f in SLE BM-MSCs might provide a potential therapeutic intervention for lupus patients. It is possible that TGF-β derived from BM-MSCs might involve in the regulation of Treg/Th17 by let-7f (26). However, our data showed that ectopic expression of let-7f had no effect on TGF-β or TGF-BR1 mRNA level by RT-PCR (Figure 2D, Supplementary Figure 3A). In addition, luciferase report demonstrated that let-7f could not regulate TGF-β expression through binding to the TGF-β1 3′-UTR (Supplementary Figure 3B), suggesting TGF-β is not the direct target of let-7f in this study. Correspondingly, both IL-6 and TGF-β are involved in T cell regulation and SLE pathogenesis, and these cytokines are actually driven by different mechanisms.

IL-6 is a pleiotropic cytokine with various biological activities in immune regulation, hematopoiesis, inflammation, and oncogenesis (32). IL-6 induces the expression of IL-23R and RORγt, thus promoting the generation of Th17 cells from naïve T cells together with TGF-β while inhibits TGF-β induced Treg (iTreg) differentiation (33). It has been reported that IL-6 levels are increased in various autoimmune diseases including rheumatoid arthritis (RA), multiple sclerosis (MS) and lupus (34), and targeting IL-6 can be an effective approach in the treatment of several autoimmune diseases (35, 36). Here we demonstrated for the first time that increased IL-6 might be induced by impaired let-7f in SLE BM-MSCs, which might led to IL-6-STAT3-NF-κB pro-inflammatory circuit, and thus contribute to lupus development.

In conclusion, in the present study we showed for the first time that reduced let-7f impairs the proliferation rate of BM-MSCs as well as BM-MSC-mediated downregulation of Th17 cells and upregulation of Treg cells but increases the apoptosis rate of BM-MSCs through targeting IL-6 and activating STAT3 pathway.

## Materials and Methods

### Patients and Controls

Totally nine active SLE patients (aSLE), six inactive SLE patients (iSLE), eight primary Sjögren's syndrome patients (pSS), five rheumatoid arthritis patients (RA), four undifferentiated connective tissue disease patients (UCTD), and ten normal subjects (NOR, mean age of 38 ± 6 years) were included in the BM-MSCs study as previously described (15). BM-MSCs and peripheral blood mononuclear cells (PBMCs) from aSLE (*n* = 9), iSLE (*n* = 6), RA(*n* = 5), pSS(*n* = 8), UCTD (*n* = 4) were used for RT-PCR assay. The demographic and clinical characteristics of these patient samples are presented in Supplementary Tables 1–3. All participants were given written consent to the study approved by the Ethics Committee of the Affiliated Drum Tower Hospital of Nanjing University Medical School.

### Antibodies and Reagents

The following antibodies (to human) were from BD Biosciences (Fremont, CA, USA): fluorescein isothiocyanate conjugated anti-human CD4, phycoerythrin (PE)-conjugated anti-human CD4 and IL-17A, allophycocyanin (APC)-conjugated anti-human CD25, phycoerythrin-Cy7-conjugated anti-human IFN-γ, IL-4, and their isotype-matched control antibodies (mouse IgG1, mouse IgG2a). The following antibodies were from eBioscience (San Diego, CA, USA): PE-Cy7-conjugated Foxp3. Purified anti-human CD3, anti-humanCD28, anti-human IL-6 antibodies and recombinant IL-6 antibodies were all from R&D Systems (Minneapolis, MN, USA). AKT inhibitor (GSK690693), p38/MAPK inhibitor (SB203580) and STAT3 inhibitor (NSC74859) were from Sigma-Aldrich (St. Louis, MO, USA). Human IL-6, TGF-β ELISA kits were from BioLegend (San Diego, CA, USA). The following antibodies (to human) were from Cell Signaling technology (Inc., USA): STAT3, AKT, MAPK, p38, p-ERK1/2, p-MAPK, p-AKT, p-p38. The following antibodies (to human) were from Proteintech (Inc., China): ERK1/2, ERK1/2, p-STAT3, and GAPDH.

### BM-MSCs Culture and miRNA Transfection

Bone marrow mononuclear cells were isolated resuspended, and cultured from all the patients and healthy controls as previously described (15). Let-7f scrambled control oligonucleotide sequence (Scrambled), let-7f-mimics oligonucleotide sequence (Let-7f-mimics) and anti-let-7f oligonucleotide sequence (Let-7f-inhibitor) were purchased from Invitrogen (Life Technologies, USA). Transient transfection of these RNA into passage 4 BM-MSCs was performed by electroporation using 4D-nucleofector™ system (Lonza, Germany) according to the manufacturer's instructions.

### Isolation and Culture of PBMCs

PBMCs were isolated from active lupus patients and healthy controls by Ficoll-Paque gradient centrifugation (Takara, Dalian, China), then were co-cultured with or without pre-plated let-7f-transfected BM-MSCs at the ratio of 10:1 in 96-well flat-bottomed plates in the presence of soluble anti-human CD3 (1 μg/ml) and anti-human CD28 (1 μg/ml) antibodies at a final volume of 200 μl RM1640 medium (Gibco, USA). In some experiments, Recombinant human IL-6 (50 ng/ml) or anti-human IL-6 antibody (30 μg/ml) were added to neutralize cytokine, and transwell system (0.4 mM pore size, Millipore) was used to block cell–cell contact. After 3-days culture, cells were harvested for examining by flow cytometry.

### Plasmid Construction and Dual Luminescence Assay

DNA fragments of IL-6 with or without 3′-UTR (WT-IL-6 3-UTR: UGGAAAGTGTAGGCTTACCTCAAATGGCT; Mut-IL-6 3-UTR: UGGAAAGTGTAGGCTTTCGTGAAATGGCT) extracted from cDNA library were cloned into psiCHECK^TM^-2 luciferase vector (Promega, USA) (Figure 5A). The recombinant control vector without the IL-6 3′-UTR was used as a negative control vector. Scrambled sequence, Let-7f-mimics, or Let-7f-inhibitor at a final concentration of 100 nM were co-transfected with the IL-6 vector or a negative control vector (1 μg/μl) using the lipofectamine 2000 (Invitrogen, USA) into HEK293 T cells. After 48 h, cells were washed and lysed with passive lysis buffer (Promega, USA), and firefly-luciferase (f-luc) and renilla luciferase (r-luc) activities were determined using the dual-luciferase reporter assay system (Promega, USA). Relative reporter activity was obtained by normalization to the f-luc activity.

### Cells Proliferation and Apoptosis Assay

Briefly, for the proliferation assay, 1 × 10^6^ cells/ml let-7f transfected BM-MSCs were incubated with 3 μM carboxyfluorescein diacetate succinimidyl ester (CFSE, Invitrogen, Camarillo, CA, USA). For the apoptosis assay, let-7f-transfected BM-MSCs (1 × 10^6^/well) were stained with Annexin-V/7AAD apoptosis detection kit Annexin V (BD Biosciences). After culturing for 4–5 days, cells were harvested for the examination by FCM, and then were analyzed by *Flowjo v10.0.7*.

### Real-Time Reverse Transcription Polymerase Chain Reaction (RT-PCR) Analysis

Total RNAs in passage 4 BM-MSCs were extracted using Trizol Reagent (Invitrogen, Carlsbad, CA, USA) according to the manufacturer's instructions. RNA integrity was determined using formaldehyde denaturalization agarose gel electrophoresis. RNA concentrations were measured by the smartspec^TM^ plus spectrophotometer (BIO-RAD, Hercules, CA, USA). cDNA was generated using SuperScript III First Strand Synthesis SuperMix (Takara, Dalian, China) or a TaqMan MicroRNA Reverse Transcript Kit (Applied Biosystems). TaqMan probes for individual miRNAs were purchased from Applied Biosystems. β-Actin and U6 was used as internal housekeeping controls, respectively. Quantification of mRNA and mature miRNA was performed on an ABI 7500 real-time PCR detection system (Applied Biosystems, USA) as previously described. The specific primers oligonucleotides (TaKaRa, Dalian, China) were used and relative expression of the target genes was calculated with the 2^−ΔΔCt^ method.

### Flow Cytometry (FCM), Western Blot and Enzyme-Linked Immunosorbent Assay (ELISA)

For FCM analysis, PBMCs were resuspended in PBS containing 1% bovine serum albumin and 0.1% sodium azide. For the staining of surface antigens, cells were incubated with FITC-, PE-, PE-Cy7-, or APC-conjugated monoclonal antibodies or their negative control antibodies as indicated for 30 min on ice. For western blot assay, we used antibodies recognizing human ERK1/2, STAT3, p38, MAPK, AKT, and their phosphorylation forms, p-ERK1/2, p-STAT3, p-p38, p-MAPK, p-AKT, and GAPDH to examine the concentrations of proteins in BM-MSCs lysates. For ELISA assay, the amounts of IL-6 and TGF-β were measurement in the conditioned media of BM-MSCs with ELISA Kits according to the manufacturer's instructions.

### Statistical Analysis

Error bars indicate SEM unless otherwise specified in the figure legend. Two-tailed unpaired Student's *t-test* were used for parametric data analysis and the Mann–Whitney U test were used for non-parametric data analysis. Comparisons among three or more groups were conducted using one-way ANOVA, and then followed by Bonferroni test among different groups. Data were calculated using the GraphPad Prism 5 software and a value of *P* < 0.05 was considered statistically significant. Statistical tests used are listed in the captions of each figure (^*^P < 0.05; ^**^P < 0.01; ^***^P < 0.001).

## Data Availability Statement

All datasets generated for this study are included in the article/Supplementary Material.

## Ethics Statement

The studies involving human participants were reviewed and approved by the Ethics Committee of the Affiliated Drum Tower Hospital of Nanjing University Medical School. The patients/participants provided their written informed consent to participate in this study.

## Author Contributions

LS and XF designed, coordinated, and supervised the study. LG and XT carried out most of the experiments. LG performed data acquisition and analysis, and wrote the manuscript. XF contributed to data interpretation and manuscript drafting. YS, SW, and DW participated in study design. YS and SW participated in human sample collection. LS, XF, BT, and LG revised the manuscript. All authors read and approved the final manuscript.

### Conflict of Interest

The authors declare that the research was conducted in the absence of any commercial or financial relationships that could be construed as a potential conflict of interest.
