# Supplementary material for: Reduced Let-7f in Bone Marrow-Derived Mesenchymal Stem Cells Triggers Treg/Th17 Imbalance in Patients With Systemic Lupus Erythematosus
Source: Front Immunol. 2020 Feb 18;11:233. doi: 10.3389/fimmu.2020.00233 (PMC7040072; doi:10.3389/fimmu.2020.00233)
Supplement: Supplementary file 1 [file Presentation_1.pdf]

Supplementary table 1. Demographic and clinical characteristics of SLE patients.

No./Age/ Sex	Disease duration , months	SLED AI score	Proteinuria (mg/24h)	Cr(μmol/L)	Clinical manifestations	Treatment
SLE1/34/F	24	6.0	580	80.6	Nephritis, polyserositis,cytopenia.	Pred, HCQ, MMF.
SLE2/22/F	12	16.0	2650	239.5	Nephritis, polyserositis, arthralgia, cytopenia, skin vasculitis.	Pred, CTX, HCQ, LEF.
SLE3/38/F	64	8.0	643	83.8	Nephritis, cytopenia, skin vasculitis	Pred, CTX, HCQ, Cellcept.
SLE4/42/F	108	10.0	3580	148.2	Nephritis, polyserositis, arthralgia ,cytopenia,	Pred, AZA, CTX, IVIG.
SLE5/32/F	120	9.0	1748	120.8	Nephritis, arthralgia, Cytopenia	CTX, LEF.
SLE6/37/F	84	13.0	1280	66.4	Nephritis, cytopenia, skin vasculitis, Neuropsychiatric involvement.	Pred, CTX.
SLE7/40/F	60	6.0	420	48.0	Arthralgia, cytopenia, skin vasculitis	Pred, HCQ, MMF.
SLE8/42/F	184	7.0	-	91.6	Cytopenia, skin vasculitis.	Pred, AZA, HCQ.
SLE9/28/F	122	10.0	1200	78.2	Nephritis, arthralgia, cytopenia, skin vasculitis.	Pred, CTX, HCQ.
SLE10/23/F	4	2	-	32	Cytopenia, skin vasculitis.	CTX.
SLE11/35/F	36	1	-	43	Cytopenia.	CTX, HCQ.
SLE12/27/F	10 day	2	-	25	Cytopenia, skin vasculitis.	-
SLE13/33/F	132	2	-	19	Arthralgia, cytopenia.	Pred, CTX, Cellcept.
SLE14/35/F	3	1	-	48	Cytopenia.	HCQ.
SLE15/46/F	192	3	-	67	Arthralgia, cytopenia, skin vasculitis	Pred, MTX, HCQ.

SLE: systemic lupus erythematosus; SLEDAI: SLE disease activity index; Cr: creatinine; F: female; Pred, Prednisone; CTX, cyclophosphamide; HCQ, hydroxychloroquine; MTX, methotrexate; MMF, mycophenolate mofetil; AZA, azathioprine; LEF, leflunomide.

Supplementary table 2. Demographic and clinical characteristics of pSS patients.

No./Age/ Sex	Disease duration, months	SSDAI score	Clinical manifestations	Drug treatment
pSS1/38/F	14	3.0	Cytopenia.	Pred, HCQ.
pSS2/51/F	72	8.0	Arthralgia, cytopenia.	Pred, NSAIDs, HCQ.
pSS3/39/F	60	9.0	Renal involvement	Pred, MMF.
pSS4/51/F	188	10.0	Arthralgia, fever.	Pred, MTX, HCQ.
pSS5/28/F	4	6.0	Cytopenia, interstitial pneumonitis.	Pred, AZA, HCQ.
pSS6/45/F	10	8.0	Arthralgia, Cytopenia.	Pred, MTX, HCQ.
pSS7/43/F	12	9.0	Arthralgia, Cytopenia.	Pred, LEF, HCQ.
pSS8/33/F	6	5.0	Cytopenia.	Pred, HCQ.

SS: primary Sjögren's syndrome; SSDAI: SS disease activity index; F: female. Pred, Prednisone; HCQ, hydroxychloroquine; MTX, methotrexate; MMF, mycophenolate mofetil; AZA, azathioprine; LEF, leflunomide; NSAIDs, nonsteroidal anti-inflammatory drugs.

Supplementary table 3. Demographic and clinical characteristics of RA and UCTD patients.

No./Age/ Sex	Disease duration, months	Clinical manifestations	Treatment
RA1/45/F	24	Arthralgia, interstitial pneumonitis.	Pred, triptolide.
RA2/41/F	12	Arthralgia.	Pred, MTX.
RA3/39/F	60	Arthralgia.	NSAIDs, MTX.
RA4/52/F	18	Arthralgia.	Pred, LEF.
RA5/32/F	64	Fever.	Pred, MTX.
UCTD1/27/F	32	Cytopenia, interstitial pneumonitis.	Pred, triptolide, HCQ.
UCTD2/32/F	38	Arthralgia, skin vasculitis.	Pred, MTX, thalidomide.
UCTD3/39/F	42	Cytopenia, skin vasculitis.	Pred, AZA, thalidomide.
UCTD4/33/F	45	Arthralgia, interstitial pneumonitis.	Pred, triptolide.

RA: Rheumatoid arthritis; UCTD, Undifferentiated connective tissue disease; F: female. Pred, Prednisone; CTX, cyclophosphamide; HCQ, hydroxychloroquine; MTX, methotrexate; MMF, mycophenolate mofetil; AZA, azathioprine; LEF, leflunomide; NSAIDs, nonsteroidal anti-inflammatory drugs.

Supplementary table 4. Correlation between expression levels of 6 abnormally expressed miRNAs and clinical characteristics of SLE patients.

Clinical characteristics	r vs p	miR-663	miR-214	miR-574-3p	miR-638	let-7f	miR-374a
SLEDAI	r	0.71	0.34	0.38	0.75	-0.70	0.39
	p	0.03	0.21	0.09	0.12	0.03	0.13
Age	r	0.22	0.04	-0.02	0.15	0.28	-0.16
	p	0.58	0.89	0.78	0.49	0.47	0.51
Duration of disease	r	0.02	0.03	0.06	0.26	0.02	-0.45
	p	0.97	0.94	0.70	0.88	0.95	0.74
C3	r	-0.68	-0.14	0.25	0.55	0.44	-0.32
	p	0.045	0.60	0.66	0.12	0.24	0.16
C4	r	-0.55	0.03	-0.15	0.36	-0.03	-0.29

	p	0.13	0.91	0.58	0.35	0.95	0.42
24h proteinuria	r	0.17	0.03	0.39	0.30	-0.67	0.59
	p	0.65	0.67	0.38	0.17	0.047	0.08
Cr	r	0.54	0.05	0.19	0.26	-0.12	0.28
	p	0.14	0.76	0.45	0.20	0.62	0.63
BUN	r	0.59	0.05	0.19	0.14	-0.07	-0.1
	p	009	0.84	0.36	0.52	0.83	0.61
UA	r	0.12	0.14	-0.21	0.83	0.10	0.55
	p	0.67	0.59	0.32	0.24	0.56	0.08
GFR	r	0.23	0.09	0.18	0.13	-0.23	-0.36
	p	0.47	0.76	0.34	0.42	0.85	0.10
CRP	r	0.75	0.06	0.36	0.67	-0.43	-0.39
	p	0.02	0.75	0.07	0.05	0.26	0.33
ESR	r	0.27	0.16	0.31	0.21	-0.66	0.09
	p	0.16	0.42	0.14	0.21	0.047	0.60
WBC	r	0.20	0.14	0.16	0.55	0.12	-0.15
	p	0.13	0.49	0.62	0.74	0.67	0.65
HB	r	0.34	0.15	0.22	0.38	-0.02	-0.09
	p	0.30	0.45	0.22	0.05	0.43	0.92
PLT	r	0.45	0.02	0.09	0.04	0.71	0.34
	p	0.09	0.92	0.68	0.86	0.03	0.24

The r and p values of nonparametric analysis are listed. SLEDAI: SLE disease activity index; yrs: years; C3: complement 3; C4: complement 4; 24h proteinuria:24-hour proteinuria; Cr: creatinine; BUN: urea nitrogen; UA: uric acid; GFR: glomerular filtration rate; CRP: C-reactive protein; ESR: erythrocyte sedimentation rate; WBC: white blood cells; HB: hemoglobin; PLT: platelet.
