# Supplementary material for: Reduced Let-7f in Bone Marrow-Derived Mesenchymal Stem Cells Triggers Treg/Th17 Imbalance in Patients With Systemic Lupus Erythematosus
Source: Front Immunol. 2020 Feb 18;11:233. doi: 10.3389/fimmu.2020.00233 (PMC7040072; doi:10.3389/fimmu.2020.00233)
Supplement: Supplementary file 2 [file Presentation_2.pdf]

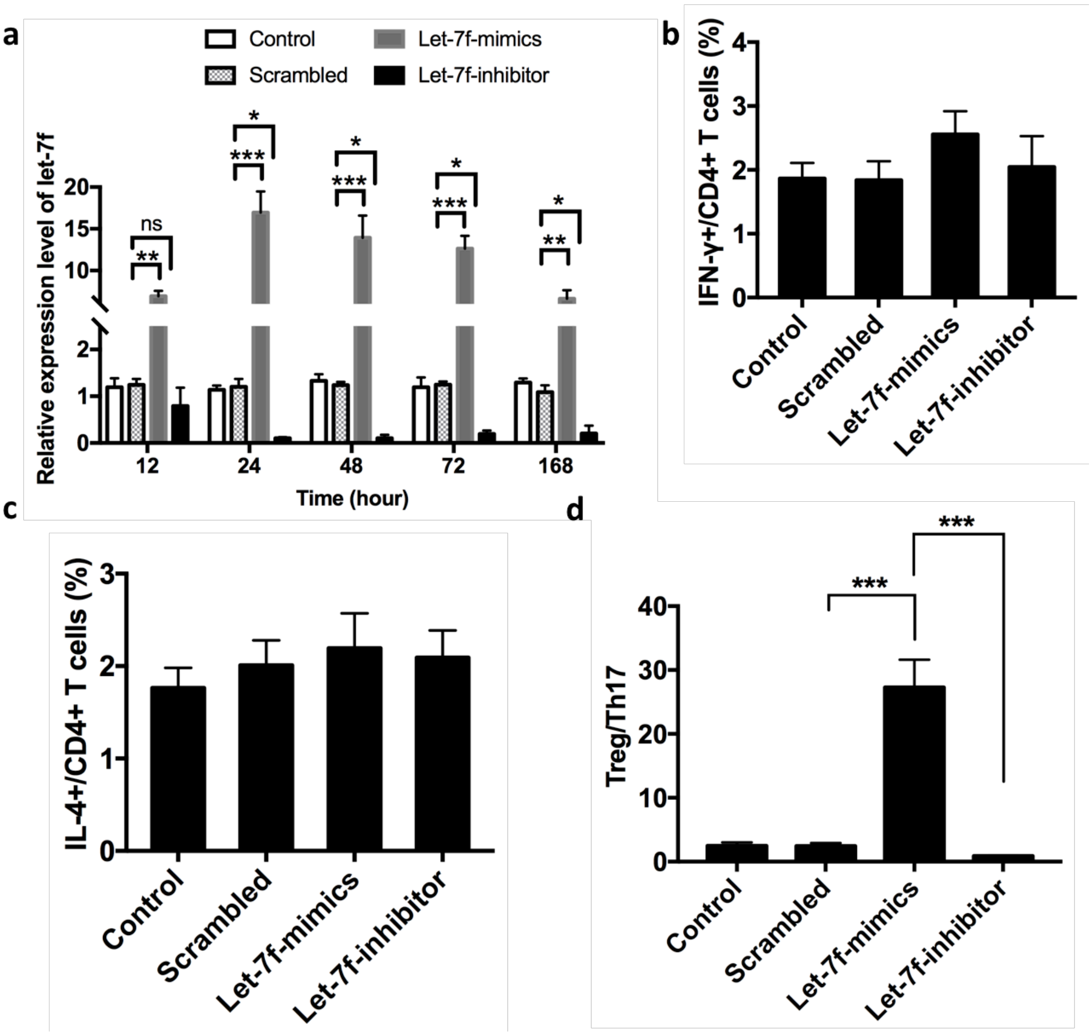

Supplementary figure 1. The role of let-7f in BM-MSCs from healthy subjects. (a) Expression levels of let-7f at 12, 24, 48, 72 and 168 hours in let-7 related oligonucleotide transfected BM-MSCs. (b-c) Proportions of Th1 cells (b, IFN-γ^{+}/$CD4^{+}$ T cells) and Th2 cells (c, IL-4^{+}/$CD4^{+}$ T cells) after co-culturing of pre-stimulated PBMCs with let-7f-related BM-MSCs. (d) The ratio of Treg ($CD25^{+}Foxp3^{+}$/$CD4^{+}$ T cells) to Th17 (IL-$17A^{+}$/$CD4^{+}$ T cells) after co-culturing of pre-stimulated naïve T cells with let-7f-related BM-MSCs. All data are means ± SEM. n=6. *$p < 0.05$, **$p < 0.01$, ***$p < 0.001$.

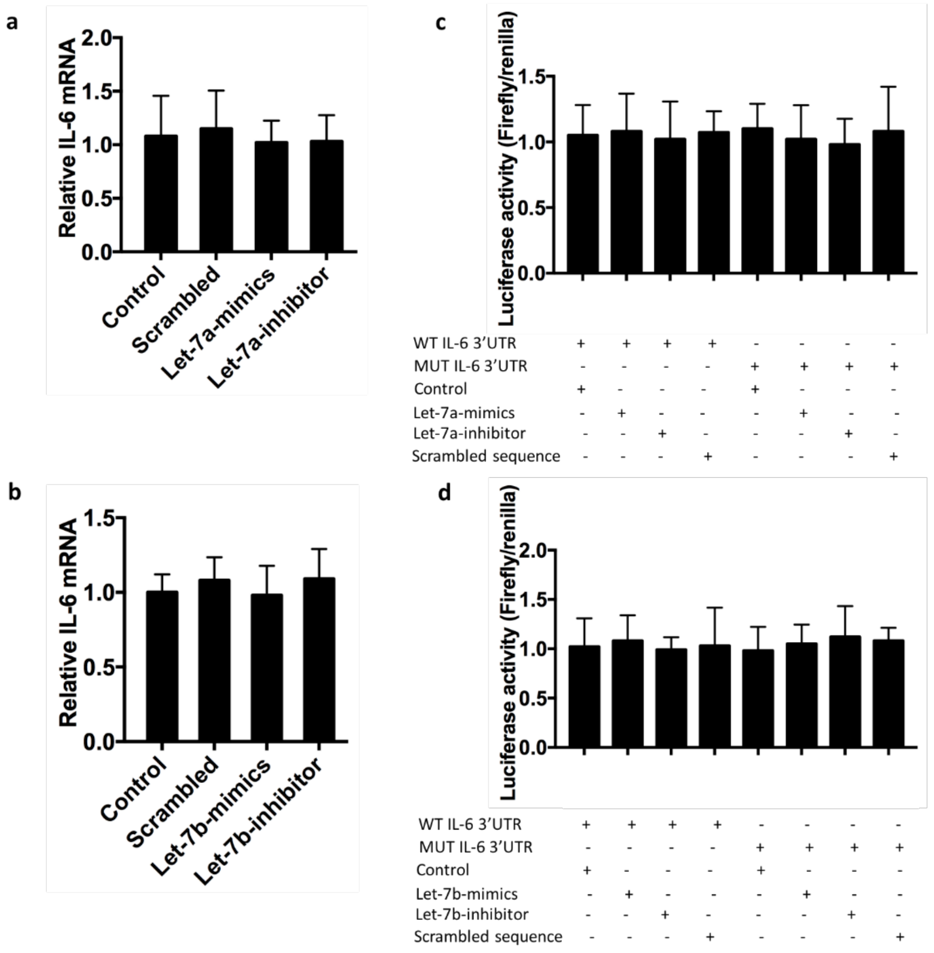

Supplementary figure 2. (a, b) IL-6 mRNA levels in various let-7a (a) or let-7b (b) related BMSCs. (c, d) The IL-6 3′-UTR is not involved in let-7a (c) and let-7b (d) regulated gene expression. All data represent the mean ± SEM. n = 6, *P<0.05, **P<0.01, ***P<0.001.

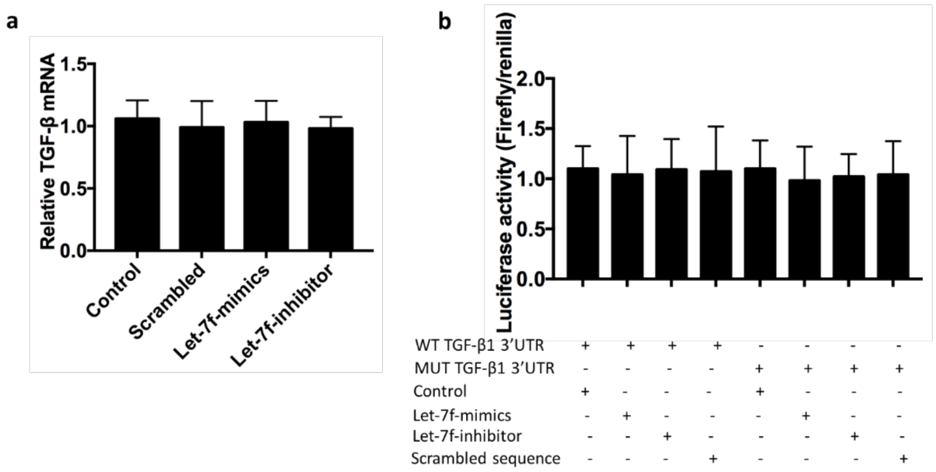

Supplementary figure 3. (a) TGF-β mRNA levels in various ectopic let-7f oligonucleotide sequence transfected BM-MSCs. (c, d) The TGF-β 3′-UTR is not involved in let-7f regulated gene expression. All data represent the mean ± SEM. n = 6.
